# Sodium citrate ingestion protocol impacts induced alkalosis, gastrointestinal symptoms, and palatability

**DOI:** 10.14814/phy2.14216

**Published:** 2019-10-10

**Authors:** Charles S. Urwin, Rodney J. Snow, Liliana Orellana, Dominique Condo, Glenn D. Wadley, Amelia J. Carr

**Affiliations:** ^1^ Centre for Sport Research, School of Exercise and Nutrition Sciences, Faculty of Health Deakin University Melbourne Victoria Australia; ^2^ Institute for Physical Activity and Nutrition, School of Exercise and Nutrition Sciences, Faculty of Health Deakin University Melbourne Victoria Australia; ^3^ Biostatistics Unit, Faculty of Health Deakin University Geelong Victoria Australia

**Keywords:** Buffering agents, dietary supplementation, ergogenic aid, ingestion mode

## Abstract

To compare the effect of 500 mg·kg^−1^ body mass (BM) sodium citrate ingested in solution or capsules on induced alkalosis, gastrointestinal symptoms and palatability. Twenty‐four healthy and active participants completed two testing sessions, ingesting 500 mg·kg^−1^ BM sodium citrate within solution or capsules. Capillary blood samples were collected pre‐ingestion, and every 30‐min for 240‐min post‐ingestion; samples were analyzed for blood pH and [HCO_3_^−^]. A validated questionnaire was used to quantify gastrointestinal symptoms at the same 30‐min intervals. Palatability was quantified immediately after ingestion using a validated scale. There was a greater peak and change from baseline for capsules versus solution for blood pH (*P* < 0.001) and [HCO_3_^−^] (*P* = 0.013). Blood pH and [HCO_3_^−^] time to peak was 199 and 204 min, respectively, after capsule ingestion, both significantly later than after solution (*P* = 0.034, *P* = 0.001). Gastrointestinal symptoms were significantly elevated above baseline for both ingestion modes at each time point between 30 and 120 min after ingestion (*P* = 0.003), with no differences between modes at any time point (*P* = 0.644). Capsules were significantly more palatable than solution (*P* < 0.001). We recommend 500 mg·kg^−1^ BM sodium citrate ingestion in capsules, at least 200 min before exercise, to achieve greater alkalosis, minimize gastrointestinal symptoms, and maximize.

## Introduction

Sodium citrate has been reported to improve the performance of short duration, high‐intensity exercise in some (McNaughton 1990; Linossier et al. 1997), but not all studies (Schabort et al. 2000; Oopik et al. 2008), raising doubts regarding its ergogenic efficacy. The reasons for the inconsistent results across previous studies are unclear, but may be attributable to suboptimal ingestion protocols, which may not allow sufficient time for peak blood alkalosis (increased blood pH, typically coinciding with an increased blood bicarbonate concentration ([HCO_3_^−^])) to occur (Urwin et al. (2016)), potentially limiting the ergogenic benefit (Potteiger et al. 1996). Further, GI symptoms such as nausea and bloating have been reported after sodium citrate ingestion (Urwin et al. 2016) and may limit the performance of affected participants (Oopik et al. 2004). Therefore, there is a need to identify an ingestion protocol which induces alkalosis and minimizes GI symptoms with the aim of improving high‐intensity exercise performance.

The most effective sodium citrate dose (500 mg·kg^−1^ body mass (BM)) has been established based on induced alkalosis, gastrointestinal, and performance outcomes (McNaughton 1990; Urwin et al. 2016); however, induced alkalosis after sodium citrate ingestion may be further affected by ingestion mode. Recent findings indicated that sodium bicarbonate ingested in polymer‐coated, gastro‐resistant capsules elicited a significant delay in peak alkalosis compared to the same dose ingested in a solution (Hilton et al. 2019), as well as a lower incidence and severity of GI disturbances (Hilton et al. 2019). The results of this investigation may be partially due to the use of the delayed‐release capsules; however, these findings may indicate that there is a difference between ingestion of buffering agents in capsules and solution.

A higher palatability for sodium citrate may increase the likelihood that, in a practical context, athletes will adhere to supplementation protocols prior to competition. While palatability cannot be directly measured, it has been established that validated scales provide a rating of participants’ overall sensory experiences, and their level of preference for a food or fluid (Yeomans, 1998). One investigation administered sodium citrate in a solution (dissolved in water) (Bracken et al. 2005) which has a salty taste, and it has been reported that foods or fluids that are high in salt can have reduced palatability (Bolhuis et al. 2011; Bolhuis et al. 2012). Some investigations have used other modes of sodium citrate ingestion, such as flavored solutions (e.g., sweetened sports drinks) (Tiryaki and Atterbom 1995; Urwin et al. 2016) or capsules (Van Montfoort et al. 2004; Vaher et al. 2015), in an attempt to modify the taste of the supplement. To our knowledge, no previous study has monitored sodium citrate palatability when comparing ingestion modes.

The primary aim of the current study was to compare the effect of 500 mg·kg^−1^ BM sodium citrate when administered via two different modes (solution or capsules) on blood alkalosis (blood pH and [HCO_3_^−^]) and GI symptoms over a 240‐min post‐ingestion period, as well as palatability immediately after ingestion.

## Materials and Methods

Physically active participants (*n* = 24; 13 males and 11 females; age, 23.4 ± 3.2 years; body mass, 73.5 ± 12.1 kg; height, 174.2 ± 9.1 cm; VO_2peak_, 45.7 ± 4.4 mL·kg·min^−1^) were recruited. Participants provided their written consent prior to experimental testing. The Deakin University Human Research Ethics Committee approved all protocols (2017‐166).

Within a randomized, cross‐over design, participants attended three testing sessions at Deakin University (Melbourne, Victoria, Australia). The first testing session included assessment of height (cm), body mass (kg), and maximal aerobic capacity (VO_2peak_) using a modified version of a previously implemented protocol (Hawley and Noakes, 1992).

### Experimental testing sessions

Participants completed a 24‐h food and activity diary, providing details of all food and fluid ingested as well as the type, duration, and intensity of exercise performed. Participants arrived at the laboratory following an overnight fast commencing at 10:00 pm the previous night.

Participants ingested 500 mg·kg^−1^ BM sodium citrate over a 30‐min period either in gelatine capsules (Melbourne Food Ingredient Depot, Melbourne, Australia) co‐ingested with 750 mL of a sports drink (Powerade, Coca Cola, USA), or diluted in the 750 mL of sports drink as a solution. A 750 mL fluid volume was included, as volumes exceeding 800 mL have previously been reported to induce a slowed gastric emptying rate (Costill and Saltin 1974; Mitchell and Voss 1991), potentially resulting in GI disturbances. Each dose was administered as three equal portions, at approximately 15‐min intervals, with ingestion completed 30 min after commencement. The mean (±SD) number of capsules ingested per participant was 36 (±6). A carbohydrate‐rich meal (1.75 g·kg^−1^ BM (Thomas and Erdman 2016)) was co‐ingested, as reduced GI symptoms have been reported with the co‐ingestion of buffering agents with carbohydrate (Price and Cripps 2012). The mean (±SD) washout period between sessions was 9 (±6) days.

Capillary blood samples were collected from the fingertip at baseline, and at 30‐min intervals for 240 min after sodium citrate ingestion commenced, and were analyzed for blood pH and [HCO_3_^−^] as previously described (Urwin et al. 2016). Participants completed a validated GI symptoms questionnaire (see Appendix S1) concurrent with capillary blood sampling (Adam et al. 2005). Sodium citrate palatability was quantified using a validated 9‐point hedonic scale (see Appendix S2) immediately post‐ingestion (Peryam and Pilgrim 1957), with participants rating the extent to which they liked ingesting sodium citrate on a 9‐point scale from 1 = dislike extremely to 9 = like extremely.

### Data management

Peak pH and [HCO_3_^−^] was defined as the maximum recorded across the 240‐min post‐ingestion period and the time to peak as time since ingestion. When change across at least three consecutive time points was negligible, the peak was defined as the first value. GI symptoms frequency was determined as the number of participants reporting each symptom at any time point.

### Statistical analyses

Stata v15 was used for all analyses. Linear mixed models (LMM) including ingestion mode and order as fixed effects and participant as random effect were fitted. For peak and change from baseline for blood pH and [HCO_3_^−^], the model also included the baseline value as a fixed effect. To compare ingestion modes for blood pH, [HCO_3_^−^] and GI symptoms across time points, the LMM included time and interaction time by mode. Sidak's method was used to adjust for multiple comparisons. Total number of GI symptoms and palatability scores were compared between ingestion modes using Wilcoxon paired tests. To compare the frequency with which each GI symptom was reported according to ingestion mode, a generalized estimating equation model with binomial distribution and logit link to account for the matched observations within participant was used. *T*‐tests compared palatability for each ingestion mode as the first vs second treatment.

## Results

**Figure 1:**
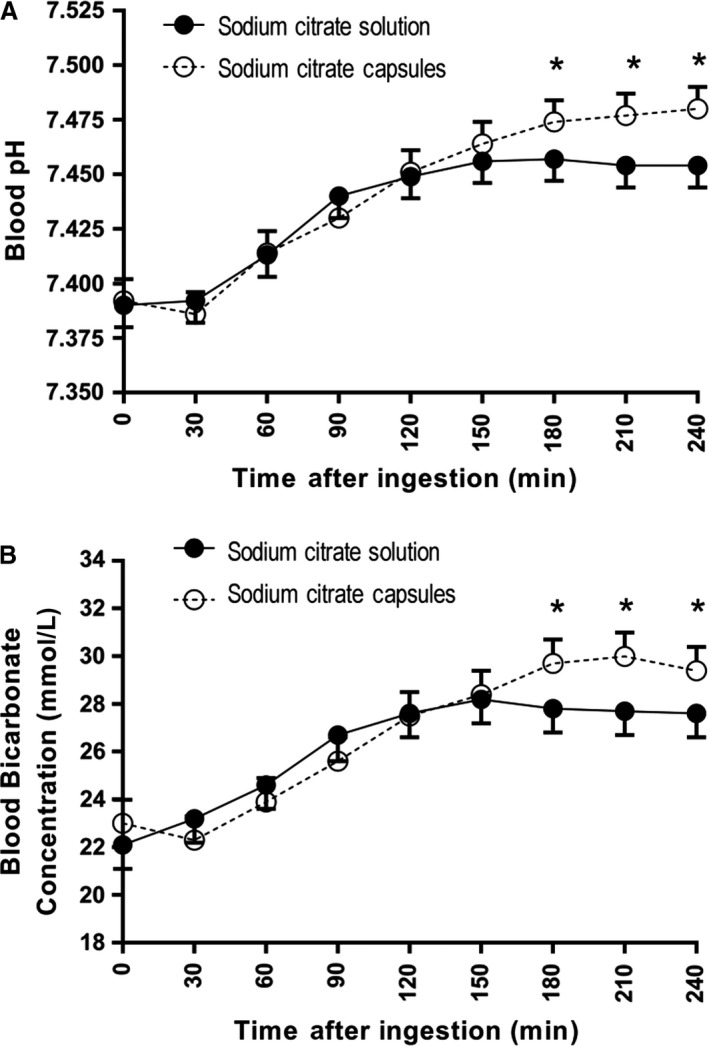
(A) Mean (95% confidence interval) blood pH for each time point following the commencement of ingestion of 500 mg·kg^−1^ body mass (BM) sodium citrate in solution or capsules (*n* = 24). Estimates obtained under a linear mixed model. * indicates *P *< 0.05, significant difference ingestion modes at the relevant time point, adjusted for multiple comparisons. (B) Mean (95% confidence interval) blood bicarbonate concentration ([HCO_3_^−^]) for each time point following the commencement of ingestion of 500 mg·kg^−1^ BM sodium citrate in solution or capsules (*n* = 24). Estimates obtained under a linear mixed model. * indicates *P *< 0.0005, significant between ingestion modes at the relevant time point, adjusted for multiple comparisons.

Blood pH and [HCO_3_^−^] significantly increased from baseline to peak (*P *< 0.001) for both capsules and solution. When comparing capsules and solution, peak blood pH (*P *< 0.001) and [HCO_3_^−^] (*P* = 0.013) were significantly greater after capsule ingestion (Table 1). Capsule ingestion elicited a significantly greater change from baseline to peak for blood pH (*P *< 0.001) and [HCO_3_^−^] (*P* = 0.013). Baseline [HCO_3_^−^] was significantly greater prior to capsule ingestion when compared to baseline prior to solution ingestion (*P* = 0.022). Capsule ingestion elicited a later time to peak compared to solution in both blood pH (*P* = 0.034) and [HCO_3_^−^] (*P* = 0.001). Compared with solution, blood pH and [HCO_3_^−^] were significantly higher at the 180, 210, and 240‐min time points after capsule ingestion (*P *< 0.05, Fig. 1).

**Table 1:** Blood alkalosis, gastrointestinal symptoms, and palatability data

	Sodium citrate solution (95% CI)	Sodium citrate capsules (95% CI)	*P*‐value (between modes)
Blood pH			
Baseline	7.390 (7.377–7.402)	7.392 (7.379–7.404)	0.724
Peak*	7.472 (7.464–7.480)	7.490 (7.482–7.498)	<0.001
Change (peak – Baseline)†	0.082 (0.074–0.089)	0.100 (0.092–0.107)	<0.001
Time to peak (min)	175 (159–191)	199 (183–215)	0.034
Blood [HCO_3_^−^]			
Baseline	22.1 (21.3–23.0)	23.0 (22.1–23.8)	0.022
Peak (mmol L^−1^)*	29.3 (28.7–30.0)	30.4 (29.8–31.1)	0.013
Change (peak – baseline)†	6.8 (6.1–7.5)	7.9 (7.2–8.6)	0.013
Time to peak (min)	164 (148–180)	204 (188–220)	0.001
Gastrointestinal symptoms†			
Total session rating (Median, IQR)	5.2 (3, 9.5)	5.8 (6, 10.25)	0.644
Palatability‡			
Score	3.5 (2.8–4.1)	6.3 (5.7–7.0)	<0.001
First mode	4.2 (3.3–5.0)	6.1 (5.2–7.0)	0.007
Second mode	2.8 (1.8–3.7)	6.6 (5.6–7.6)	<0.001

Differences according to ingestion mode for blood pH and blood bicarbonate concentration ([HCO_3_^−^]): linear mixed models. Differences according to ingestion mode for gastrointestinal symptoms total ratings and palatability scores: Wilcoxon paired tests. Differences between first treatment and second treatment: independent samples *t*‐test.

Blood analysis, gastrointestinal symptoms, and palatability parameters expressed as mean (95% confidence intervals), *N *= 24. IQR, interquartile range.

* Comparison peak versus baseline.

^†^ Comparison peak value – baseline value.

^‡^ Sum of rating for all symptoms across an entire session, regardless of time point.

^§^ Comparison between participants ingesting each mode as the first treatment versus the second treatment: solution *P* = 0.025, capsules *P* = 0.425.

**Figure 2:**
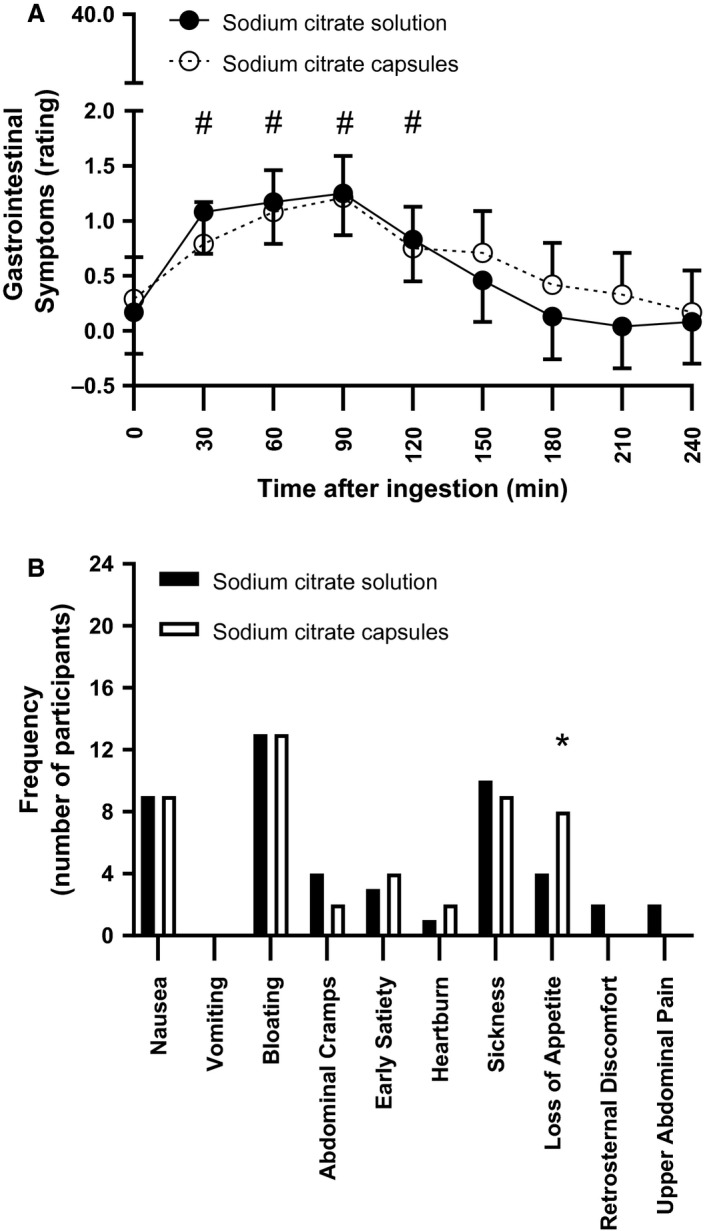
(A) Mean (95% confidence interval) of all gastrointestinal symptoms rating at each time point following the commencement of ingestion of 500 mg·kg^−1^ BM sodium citrate in solution or capsules (*n *= 24). Estimates obtained under a linear mixed model. # indicates *P *< 0.005, significant difference compared to baseline value within treatment, adjusted for multiple comparisons. (B) Frequency of gastrointestinal symptoms reported by all participants (*n* = 24). Values represent the number of participants that reported each symptom, regardless of time point or rating of that symptom, with each participant accounted for a maximum of once only. * indicates *P *< 0.05, significant difference between ingestion modes for frequency of the relevant symptom.

GI symptoms (total rating) were not significantly different between solution and capsules for total session values (*P* = 0.644; Table 1), or at any individual time point (*P* > 0.05, Fig. 2A). For both capsules and solution, GI symptoms were significantly elevated above baseline at 30, 60, 90, and 120‐min time points after ingestion (*P *< 0.005, Fig. 2A); however, the symptoms were minor, given the total session ratings of 5.2 and 5.8 out of 360 for solution and capsules, respectively. Loss of appetite was reported more frequently following the ingestion of sodium citrate in capsules compared to solution (*P* = 0.034, Fig. 2B); bloating was the most commonly reported symptom, followed by sickness and nausea (Fig. 2B). Sodium citrate capsules were rated as significantly more palatable than solution (*P *< 0.001; Table 1).

## Discussion

This study aimed to compare the effect of sodium citrate ingestion when administered via two different modes (solution vs capsules) on blood pH, [HCO_3_^−^], GI symptoms and palatability. Key findings were that ingestion of sodium citrate in capsules resulted in a significantly greater peak and change from baseline for blood pH and [HCO_3_^−^], which occurred at a later time point, and was more palatable, when compared to solution. While GI symptoms were minor after both ingestion modes, symptoms were elevated significantly above baseline at each time point between 30 and 120 min after ingestion, whereas alkalosis peaked approximately 200 min after ingestion of capsules.

To our knowledge, this is the first study to quantify the induced alkalosis after sodium citrate ingestion in capsules compared with solution. In the current investigation, there was significantly higher blood pH and [HCO_3_^−^] following capsule ingestion compared to solution at 180, 210, and 240‐min post‐ingestion time points. Peak and change from baseline to peak for both blood pH and [HCO_3_^−^] were significantly greater after capsule ingestion when compared to solution. Therefore, it can be recommended that 500 mg·kg^−1^ BM sodium citrate be ingested in capsules rather than solution, at least 180 min before exercise commencement, to facilitate a greater peak and change from baseline in alkalosis.

The higher peak blood pH and [HCO_3_^−^] following sodium citrate capsule ingestion compared with solution was not expected, given the matched dose of the supplement administered. A recent sodium bicarbonate investigation also reported a delayed peak alkalosis after capsule ingestion compared with solution (Hilton et al. 2019); however, no significant difference in the peak was reported. The reasons for the difference between solution and capsules in peak and change from baseline for blood pH and [HCO_3_^−^] in the present study are not clear, although there are some potential mechanistic explanations. After sodium citrate ingestion, alkalosis likely occurs due to the entry of its constituent ions (sodium and citrate) into the circulation via the small intestine, causing a shift in strong ion difference (SID) (Lindinger and Heigenhauser 1991), resulting in either decreased [HCO_3_^−^] excretion or increased [H^+^] excretion by the kidneys to restore homeostasis. There may be a more gradual release of sodium and citrate ions into the small intestine following capsule ingestion, due to the protein content of the capsules (Borgström et al. 1957). This may result in a greater proportion of sodium and citrate ions entering the circulation, rather than passing into feces as may occur in solution treatments. Future investigations should consider assessment of additional physiological markers that may further clarify the mechanisms by which this outcome occurred.

In the current investigation, only the capsule treatment induced a peak alkalosis that was consistent with that reported in the majority of previous investigations where both alkalosis and a performance benefit were reported (blood pH > 7.45 and [HCO_3_^−^] > 30 mmol L^−1^) (McNaughton 1990; McNaughton and Cedaro 1992; Lindh et al. 2008; Siegler and Gleadall‐Siddall 2010). Therefore, implementing the use of capsules may be a strategy that is more likely to achieve a sufficient peak alkalosis to improve exercise performance. Surprisingly, baseline [HCO_3_^−^] was significantly higher in the capsule treatment compared to solution, despite the order of administration being randomized and implementation of a washout period averaging 9 days per participant. It should be noted, however, that baseline values were included in relevant LMM analyses as fixed effects, reducing the influence of this difference on subsequent analyses.

This is the first study to compare GI symptoms after ingestion of sodium citrate via different modes. In the present investigation, rating of GI symptoms did not differ according to ingestion mode at any specific time point, or across total sessions. A limitation of the present investigation is that the experimental design prevents the identification of the factor(s) causing the small but significant increase in GI symptoms post‐ingestion, but this could be due to the sodium citrate dose, fluid volume/composition, and/or meal. All of these factors can influence gastric emptying rate and subsequent GI symptoms (Mitchell and Voss 1991; de Oliveira et al. 2014).

The results of the current study indicated that ingestion of capsules caused “loss of appetite” significantly more frequently than solution, possibly due to the greater total ingested volume and/or mass of the capsule treatment caused by the presence of the capsules, both characteristics which can slow gastric emptying rate and potentially cause GI disturbances (Hunt and Stubbs 1975; Moore et al. 1981; Kilara et al. 1998). However, further investigation is needed to clarify the impact of these characteristics, given that the difference in volume due to capsule ingestion was not monitored, and the added mass of the capsules was only approximately 3.6 g (0.1 g per capsule) per participant, which may be insufficient to influence appetite. Alternatively, the “loss of appetite” observed may have been a result of the greater protein content of the capsules compared to solution, given that protein intake is positively associated with satiety (Paddon‐Jones et al. 2008). The additional protein from the capsules, however, was relatively small, so the impact of this added protein ingestion is unclear. It should be noted that the occurrence of “loss of appetite” did not preclude any participant from completing ingestion of sodium citrate or the standardized meal. Therefore, the more frequent “loss of appetite” in the capsule treatment was not considered sufficient to limit the practical application of an ingestion protocol where sodium citrate is administered in capsules.

For both ingestion modes, GI symptoms were elevated above baseline at each time point from 30 to 120 min after ingestion, consistent with a previous investigation within our laboratory, where peak GI symptoms occurred within the same post‐ingestion period (83‐min post‐ingestion) (Urwin et al. 2016). The minor nature of GI symptoms both in the present study and the recent dose–response study (Urwin et al. 2016) suggest that a low incidence of GI symptoms following 500 mg·kg^−1^ BM sodium citrate ingestion is possible when administered via capsules, co‐ingested with a carbohydrate‐rich meal. Therefore, allowing a period of at least 120 min between ingestion and exercise may decrease the likelihood of athletes experiencing GI disturbances at the start of exercise.

To the authors’ knowledge, no previous sodium citrate investigation has quantified palatability. Participants rated their levels of preference for the two sodium citrate treatments immediately post‐ingestion, which is a well‐established method of measuring palatability (Peryam and Pilgrim 1957; Yeomans 1998). An excessively salty taste may result in avoidance behaviour and low palatability scores (Bolhuis et al. 2011; Bolhuis et al. 2012), which have previously been reported when foods or fluids provide high levels of stimuli for one specific taste (bitter, sweet, sour, salty, umami) (McCrickerd and Forde 2016). The indistinct taste of gelatine capsules may therefore provide some explanation as to why capsules were the preferred mode of ingestion, and therefore more palatable than solution. The solution was rated as significantly less palatable when administered as the second treatment, when compared to the solution administered as the first treatment. This outcome suggests that participants who had previously ingested sodium citrate in capsules deemed the solution to be less palatable when compared to participants who did not have a reference point (the palatability of the capsules) with which to rate the palatability of the solution. This supports the finding that participants liked ingestion of the supplement to a greater extent in capsules when compared to solution, as indicated by a significantly higher palatability score for capsules in all analyses comparing the two ingestion modes.

The primary aim of this investigation was to compare ingestion of sodium citrate via two different modes (solution and capsules), which was addressed without the inclusion of a placebo condition. Inclusion of a placebo treatment in future sodium citrate investigations may assist in determining if the occurrence of minor GI symptoms is affected by the sodium citrate dose, fluid volume/composition, and/or co‐ingested food. It was deemed impractical to blind participants to the experimental conditions, given that it is not possible to blind participants as to whether they ingest capsules or solution within experimental testing sessions.

## Conclusion

It is recommended that sodium citrate be ingested at a dose of 500 mg·kg^−1^ BM, in gelatine capsules, a minimum of 180 min before commencement of exercise.

## Conflict of Interest

The authors declare that there is no conflict of interest. All aspects of the present investigation comply with the current laws of Australia.

## Supporting information




**Appendix S1**. Validated Gastrointestinal Symptoms Questionnaire (Adam et al. 2005). The severity of 10 different symptoms (nausea, vomiting, bloating, abdominal cramps, early satiety, heartburn, sickness, loss of appetite, retrosternal discomfort, and upper abdominal pain) were rated on the 5‐point, Likert type scale ranging from 0 – no problem to 4 – very severe problem.

**Appendix S2** **.** Validated Palatability Questionnaire (Peryam and Pilgrim 1957). Palatability of sodium citrate was quantified using a Hedonic scale, with participants rating the extent to which they liked sodium citrate on a 9‐point scale from 1 – dislike extremely, to 9 – like extremely.
