# Supplementary material for: Sodium citrate ingestion protocol impacts induced alkalosis, gastrointestinal symptoms, and palatability
Source: Physiol Rep. 2019 Oct 10;7(19):e14216. doi: 10.14814/phy2.14216 (PMC6787309; doi:10.14814/phy2.14216)
Supplement: Supplementary file 1 — Appendix S1. Validated Gastrointestinal Symptoms Questionnaire (Adam et al. 2005). The severity of 10 different symptoms (nausea, vomiting, bloating, abdominal cramps, early satiety, heartburn, sickness, loss of appetite, retrosternal discomfort, and upper abdominal pain) were rated on the 5‐point, Likert type scale ranging from 0 – no problem to 4 – very severe problem. [file PHY2-7-e14216-s001.pdf]

Gastrointestinal Symptoms Questionnaire

General Information

Name: Date:

Time: Questionnaire Number:

Symptoms

Which of the following are you experiencing **at this point in time?**

	No Problem	Mild Problem	Moderate Problem	Severe Problem	Very Severe Problem
**Nausea**	☐	☐	☐	☐	☐
**Vomiting**	☐	☐	☐	☐	☐
**Bloating**	☐	☐	☐	☐	☐
**Abdominal Cramps**	☐	☐	☐	☐	☐
**Early Satiety**	☐	☐	☐	☐	☐
**Heartburn**	☐	☐	☐	☐	☐
**Sickness**	☐	☐	☐	☐	☐
**Loss of Appetite**	☐	☐	☐	☐	☐
**Retrosternal Discomfort**	☐	☐	☐	☐	☐
**Upper Abdominal Pain**	☐	☐	☐	☐	☐

See back page of this questionnaire for definitions of the above symptoms.

Nausea – Urgent feeling of need to vomit but vomit does not actually occur

Vomiting – Vomiting of mucus and gastric contents or strong unproductive retching

Bloating – Feeling of congestion of food without relation to prior food intake which could explain this feeling

Abdominal Cramps – Spasmodic or colic-like stomach pain without specified localization

Early Satiety – Feeling that the stomach is overfilled soon after starting to rest, unproportional to the quantity of food taken, so that the meal cannot be finished

Heartburn – Belching with acid taste, burning sensation in the oesophagus

Sickness – Discomfort combined with the impression for the need to vomit

Loss of Appetite – Listless for food intake

Retrosternal Discomfort – Unpleasant feeling behind the sternum, painful or drawing

Upper Abdominal Pain – Pain localized between the costal arches (ribs), below the sternum.
