# Supplementary material for: Sodium citrate ingestion protocol impacts induced alkalosis, gastrointestinal symptoms, and palatability
Source: Physiol Rep. 2019 Oct 10;7(19):e14216. doi: 10.14814/phy2.14216 (PMC6787309; doi:10.14814/phy2.14216)
Supplement: Supplementary file 2 — Appendix S2 . Validated Palatability Questionnaire (Peryam and Pilgrim 1957). Palatability of sodium citrate was quantified using a Hedonic scale, with participants rating the extent to which they liked sodium citrate on a 9‐point scale from 1 – dislike extremely, to 9 – like extremely. [file PHY2-7-e14216-s002.pdf]

Palatability Questionnaire

Name _______________ Session ______________

Date _______________ Time ________________

Sample_________Overall evaluation. Place a mark in the box which you feel best describes how you like the product:

☐	☐	☐	☐	☐	☐	☐	☐	☐
Like extremely	Like very much	Like moderately	Like slightly	neither like nor dislike	Dislike slightly	Dislike moderately	Dislike very much	dislike extremely
